# Troponina Cardíaca como Preditor de Injúria Miocárdica e Mortalidade por COVID-19

**DOI:** 10.36660/abc.20200862

**Published:** 2020-10-13

**Affiliations:** 1 Universidade Federal do Rio de Janeiro Rio de JaneiroRJ Brasil Universidade Federal do Rio de Janeiro, Rio de Janeiro, RJ – Brasil; 2 Rede D’Or Sao Luiz Rio de JaneiroRJ Brasil Rede D’Or Sao Luiz, Rio de Janeiro, RJ – Brasil

**Keywords:** COVID-19/complicações, Betacoronavírus/complicações, Doenças Cardiovasculares, Cardiomiopatia Hipertrófica Familiar, Mortalidade, Hospitalização, Troponina T

No Brasil, até o dia 1º de agosto de 2020, foram diagnosticados 2.707.877 casos de COVID-19, com acúmulo de 93.563 óbitos. A taxa de letalidade para o país, nesse período, foi de 3,5% com mortalidade variável conforme a região estudada, sendo a menor na região Sul (11,1 mortes/100 mil habitantes) e a maior na região Norte (60,2 mortes/100 mil habitantes).^1^ Até 20% dos indivíduos infectados requerem hospitalização, e destes, cerca de 25% necessitam de cuidados em unidade de terapia intensiva (UTI).^2^ Postula-se que a ocorrência de intensa resposta inflamatória e a formação de um estado de hipercoagulabilidade, potencializadas por hipoxemia, justifique os principais achados clínicos e laboratoriais nos casos graves de infecção pelo novo coronavírus.^3^

Nessa população, a ocorrência de injúria miocárdica (IM) não é incomum, e a elevação de troponina cardíaca (cTn) I comporta-se como preditor de mortalidade intra-hospitalar.^4^ Também há possibilidade de lesão direta pelo vírus, causando miocardite.^5^ Um estudo de necrópsia que documentou a presença do vírus em 61,5% dos participantes não observou o afluxo de células inflamatórias no miocárdio na fase aguda, e a consequência a longo prazo dessa infecção cardíaca ainda não é conhecida.^6^ Todavia, a incidência de IM em pacientes hospitalizados por essa enfermidade no Brasil é pouco conhecida, e seu impacto prognóstico ainda é mal elucidado. O estudo multicêntrico com biomarcadores cardíacos é dificultado pelo emprego de diferentes testes laboratoriais entre as instituições.

O diagnóstico de IM é baseado na identificação de ao menos um valor de cTn acima da referência superior da normalidade. Isso porque variações em análises seriadas desse biomarcador sugerem lesão aguda das células cardíacas, embora não seja possível determinar o mecanismo fisiopatológico subjacente apenas pela sua mensuração. As razões para sua ocorrência podem ser agrupadas como: causas cardíacas isquêmicas, não isquêmicas e sistêmicas.^7^^,^^8^ Elevações de cTn são comuns em pacientes internados em UTI e estão relacionadas a maiores eventos adversos, independentemente da doença de base.^9^

Um estudo pioneiro, desenvolvido na cidade do Rio de Janeiro, com amostra de conveniência abrangendo 183 casos confirmados de COVID-19 internados em hospital terciário, buscou avaliar o valor prognóstico da cTn T e do peptídeo natriurético do tipo B (BNP) nessa população. Concluiu-se que a cTn T, mas não o BNP, foi um marcador independente de risco para morte intra-hospitalar ou necessidade de ventilação mecânica invasiva.^10^ Uma limitação foi a não inclusão de dados obtidos por meio de eletrocardiografia e ecocardiografia, justificada por política institucional que visava à melhor distribuição de recursos financeiros e à proteção de recursos humanos. Além disso, a cTn foi verificada apenas nas primeiras 24h de internação.

Sabe-se que o quadro inflamatório secundário à tempestade de citocinas (fase III) ocorre depois da fase puIMonar (fase II), com um intervalo médio de 5 dias. Desse modo, a dosagem seriada de cTn seria a melhor abordagem, pois auxiliaria a identificação do paciente com IM não detectada na admissão.^11^

Uma metanálise realizada em março de 2020 por Giuseppe Lippi et al. mostrou, em seus resultados, que aumentos significativos da cTn correspondendo à IM são encontrados em cerca de 8 a 12% de todos os casos de COVID-19, sendo mais frequentes nas manifestações graves da doença.^12^ Um estudo recentemente publicado, realizado com 2.736 pacientes com COVID-19, observou que a IM, quantificada pelo aumento da cTn, mesmo com baixos valores, e principaIMente naqueles com história de doença cardiovascular (DCV), foi associada a alto rico de morte.^13^

Os dados obtidos pelo estudo brasileiro^10^ reforçam a impressão já levantada por outros autores de que a elevação de cTn na COVID-19 está ligada a piores desfechos clínicos.^11^^–^^14^ Assim, à luz dos dados atuais, é possível que a utilização desse biomarcador na estratificação de risco em pacientes com COVID-19 seja uma estratégia exequível, levando-se em consideração o benefício em identificar o acometimento cardíaco sem expor outros profissionais durante a realização de exames como eletrocardiograma e ecocardiograma.

Portanto, a IM é comum em pacientes com COVID-19 e pode ser explicada por diferentes mecanismos fisiopatológicos. Até o momento, não existe recomendação para terapia específica dessa lesão por IM relacionada à infecção pelo novo coronavírus; no entanto, a dosagem de cTn ao longo da internação hospitalar pode facilitar a classificação de risco desses pacientes, com a vantagem de ser um método faciIMente reprodutível e com mínima exposição da equipe de saúde envolvida na sua execução, o que é especificamente útil ao controle de propagação viral em ambiente hospitalar.
