# Supplementary material for: Troponina Cardíaca como Preditor de Injúria Miocárdica e Mortalidade por COVID-19
Source: Arq Bras Cardiol. 2020 Oct 13;115(4):667–8. [Article in Portuguese] doi: 10.36660/abc.20200862 (PMC8386976; doi:10.36660/abc.20200862)
Supplement: Translation

# Translation: Cardiac Troponin as a Predictor of Myocardial Injury and Mortality from COVID-19

**Affiliations:** 1 Rio de JaneiroRJ Brazil Universidade Federal do Rio de Janeiro, Rio de Janeiro, RJ – Brazil; 2 Rio de JaneiroRJ Brazil Rede D’Or Sao Luiz, Rio de Janeiro, RJ – Brazil

**Keywords:** COVID-19/complications, Betacoronavirus/complicações, Cardiovascular Diseases, Cardiomyopathy, Hypertrophic Familial, Mortality, Hospitalization, Troponin T

In Brazil, until August 1, 2020, 2,707,877 cases of COVID-19 were diagnosed, with 93,563 deaths. the lethality rate for the country, in this period, was 3.5% with mortality variable mortality depending on the region studied. It is the lowest in the South (11.1 deaths/100,000 inhabitants) and the highest in the North (60.2 deaths/100,000 inhabitants).^1^ Up to 20% of infected individuals require hospitalization and, of these, about 25% need to be taken to an intensive care unit (ICU).^2^ In severe cases of COVID-19, intense inflammatory response and hypercoagulability enhanced by hypoxemia justify the main clinical and laboratory findings.^3^

In this population, the presence of Myocardial Injury (MI) is not uncommon, and increased cardiac troponin (cTn)I behaves as a predictor of in-hospital mortality.^4^ There is also a possibility of direct injury by a virus that could generate myocarditis.^5^ A necropsy study that documented the presence of a virus in 61.5% did not observe the inflow of inflammatory cells in the myocardium in the acute phase, and the long-term consequence of this cardiac infection is not yet known.^6^ However, the incidence of MI in patients admitted for this disease in Brazil is little known, and its prognostic impact is still poorly elucidated. A multicenter study with cardiac biomarkers is hampered by different laboratory tests between institutions.

Diagnosis of (MI) is based on the identification of at least one cTn value above the normal upper range. Variations in serial analyses of this biomarker suggest acute cardiac cell damage, though not being able to determine the underlying pathophysiological mechanism just by measuring it. Reasons for its occurrence can be grouped as ischemic cardiac, non-ischemic, and systemic cardiac causes.^7^^,^^8^ Increased cTn is common in ICU patients and is related to more significant adverse events regardless of the underlying disease.^9^

A pioneering study developed in Rio de Janeiro with a convenience sample of 183 confirmed cases of COVID-19 admitted to a tertiary hospital assessed the prognostic value of cTn T and BNP in this population. They concluded that cTn T, but not BNP, was an independent risk marker for in-hospital death or need for invasive mechanical ventilation.^10^ One of the study limitations was the lack of electrocardiography and echocardiography data, justified by an institutional policy aimed at better distribution of financial resources and at protecting healthcare professionals. Besides, troponin was checked only in the first 24 hours of hospitalization.

We know that the inflammatory condition secondary to cytokine storm (phase III) occurs after the pulmonary phase (phase II), with an average interval of 5 days. Thus, the serial dosage of troponin would be the best approach as it would help to identify patients with myocardial injury not detected at admission.^11^

A meta-analysis carried out in March 2020 by Giuseppe Lippi et al. found that significant increases in troponin corresponding to myocardial injury are found in about 8 to 12% of all cases of COVID-19, and is more frequent in critically ill patients.^12^ A recently published study carried out with 2,736 patients with COVID-19 found that myocardial injury, quantified by a rise in troponin, even if this is a modest rise, and especially in those with a history of cardiovascular disease, was associated with a high death rate.^13^

Data obtained by this Brazilian study^10^ reinforce the impression raised by other authors that increased cTn in COVID-19 is associated with worse clinical outcomes.^11^^–^^14^ The use of this biomarker in risk stratification in patients with COVID-19 may be a feasible strategy to identify cardiac involvement without exposing healthcare professionals to electrocardiography and echocardiography scans.

In conclusion, MI is common in COVID-19 patients and could be explained by different pathophysiological mechanisms. So far, there is no recommendation for specific IM therapy related to infection by a new coronavirus. However, measurement of cTn during hospitalization can facilitate the risk classification of these patients with the advantage of being an easily reproducible method with minimal exposure of the health team involved in its execution, which is specifically useful for controlling viral spread in a hospital environment.
